# Is there enough research output of EU projects available to assess and improve health system performance? An attempt to understand and categorise the output of EU projects conducted between 2002 and 2012

**DOI:** 10.1186/s12961-016-0165-5

**Published:** 2017-02-22

**Authors:** Britta Zander, Reinhard Busse

**Affiliations:** Department of Health Care Management, Berlin University of Technology, Berlin, Germany

**Keywords:** Health system performance assessment, EU healthcare projects, Individual-level data, Data accessibility

## Abstract

**Background:**

Adequate performance assessment benefits from the use of disaggregated data to allow a proper evaluation of health systems. Since routinely collected data are usually not disaggregated enough to allow stratified analyses of healthcare needs, utilisation, cost and quality across different sectors, international research projects could fill this gap by exploring means to data collection or even providing individual-level data. The aim of this paper is therefore to (1) study the availability and accessibility of relevant European-funded health projects, and (2) to analyse their contents and methodologies.

**Methods:**

The European Commission Public Health Projects Database and CORDIS were searched for eligible projects, which were then analysed by information openly available online.

**Results:**

Overall, only a few of the 39 identified projects produced data useful for proper performance assessment, due to, for example, lacking available or accessible data, or poor linkage of health status to costs and patient experiences. Other problems were insufficient databases to identify projects and poor communication of project contents and results.

**Conclusions:**

A new approach is necessary to improve accessibility to and coverage of data on outcomes, quality and costs of health systems enabling decision-makers and health professionals to properly assess performance.

**Electronic supplementary material:**

The online version of this article (doi:10.1186/s12961-016-0165-5) contains supplementary material, which is available to authorized users.

## Background

Adequate health system performance information is required to assess whether health systems offer high-performing health services to their populations, and therefore to carry out actions to improve them [1]. The necessary information could be collected and analysed at the national level; however, more important conclusions can be drawn through international comparisons, ideally, if individual-level data for different diseases, interventions and providers is available to take account of variations in population groups, such as health status, income and educational level, and service delivery. Thus, a better understanding of how processes may lead to various outcomes can be achieved compared to aggregated data that only offers average values. Users of the information generated by individual-level data are not only health service researchers but all those who are interested in health systems – such as epidemiologists, economists, policy advisors or funders of research. While individual expectations might differ, they optimally all expect to find (access to) data, which are individual level, longitudinal and detailed enough to allow a stratified analysis of healthcare needs, healthcare utilisations, costs and quality across different sectors and performance areas of the healthcare system, not only within individual countries, but across different countries.

However, disaggregated data are often still not routinely collected, analysed, reported and/or made available for research due to several privacy and data protection issues. Research projects could be theoretically used to fill this gap, either by exploring how individual-level data can be collected and usefully analysed or even by regularly providing such data, both within and especially across countries. In order to facilitate the search of such health information, several EU health information systems were initiated, including CORDIS (the Community Research and Development Information Service for Science, Research and Development, http://cordis.europa.eu/projects/home_en.html), the Health-EU portal (http://ec.europa.eu/health/index_en.htm), EUPHIX (EU Public Health Information & Knowledge System, http://www.euphix.org/) or DG SANCO’s HEIDI wiki (Health in Europe: Information and Data Interface) (https://webgate.ec.europa.eu/sanco/heidi/index.php/Main_Page). In reality, however, it has often remained a challenge to maintain an overview of all data produced (let alone to access them). The Health Research for Europe project, which aimed to synthesise results of health research from the EU’s Fifth and Sixth Framework Programmes (FP5 and FP6), criticised CORDIS for its insufficient data collection and compared the platform with the concept of a black hole, since “*the results of projects seem to disappear*” [2] when searching for EU-funded research [3]. EUPHIX also seems to be inadequate, since it had to be taken off-line due to discontinuity of funding.

The unavailability of adequate information thus hampers the evidence base to improve performance [3]. Furthermore, a lot of research reported in conference abstracts has never been published as full reports [4] – and to be more accurate, more than 50% of the 3691 health-related research projects funded in FP5 and FP6 did not produce any academic output that could be traced through PubMed or Google scholar [5]. Furthermore, sufficient strategies adopted by researchers to inform policymakers on their results are scarce [6]. Nevertheless, since Horizon 2020, much has moved in the right direction as the Commission recognised their need to address those imperfections [7]; however, it still remains to be asked: How much is really known about EU-funded research projects and their results?

The compartmentalisation of health data initiatives on the one hand and poor integration between EU Research Programmes and national programmes on the other, result in uncoordinated health research activities between member states [8]. To overcome this challenge, the FP7-project EuroREACH (A Handbook to Access Health Care Data for Cross-Country Comparisons of Efficiency and Quality) was initiated to contribute to a more systematic performance assessment of health systems by enabling access to and use of international and national health information systems and EU research projects. An online platform, the Health Data Navigator (www.healthdatanavigator.eu), was developed to share the output of the project online. The present article has derived from work done in one of EuroREACH’s work packages, namely WP2, which investigated international and EU health projects with the aim to improve consistency and comparability of healthcare data. Since previous research suggested to place more emphasis on the output of EU projects rather than on the inputs, we approached it yet from a different perspective with an attempt to understand and categorise the output of EU health projects in order to provide a potential user of comparative healthcare information with a data inventory to address a hypothesised research need. Thus, the following questions guide our analysis:

1. What kind of health information are we able to find with respect to the availability and accessibility of relevant output from EU-projects?
2. Are we able to identify any pattern in the distribution of research funding towards certain performance domains, care settings, disease groups or scope of research?

## Methods

We concentrated our search on EU health research projects and initiatives. Therefore, a systematic search using relevant search terms was conducted in CORDIS as well as in the European Commission Public Health Projects Database (http://ec.europa.eu/health/projects/index_en.htm). The systematic search was done in two rounds of screening in each of the databases. The search in the European Commission Public Health Projects Database was filtered through the following selectable terms: ‘major and chronic disease’, ‘mental health’, ‘health indicators’, and ‘data collection’, and by year. Projects conducted between 2000 and 2003 were searched in the archive database (http://ec.europa.eu/health/ph_projects/project_previous_en.htm), filtered by ‘health monitoring’ and ‘cancer’, since the abovementioned terms were not selectable therein. In CORDIS, the search was filtered by framework programs (5th, 6th, 7th), and refined through the generic subjects ‘medicine and health’ and ‘healthcare delivery/services’. Since the CORDIS database search does not allow the option of filtering projects by topic (e.g. by disease), the search in each framework was done by entering following keywords into the search bar: ‘asthma’, ‘cardiovascular diseases’, ‘diabetes’, ‘cancer’, ‘chronic obstructive pulmonary disease’, ‘chronic disease’, ‘mental health’, ‘equity’ and ‘quality’.

In total, 63 projects (18 in European Commission Public Health Projects Database and 45 in CORDIS) were identified on project title review and information based on the following inclusion criteria, as agreed by the EuroREACH consortium, (1) conducted between 2000 and 2012 (ongoing projects which started before 2000 were included); (2) comparative across countries (i.e. at least two countries involved); (3) using individual-level data (the one exception made to this rule was to include population-based cancer registry data); (4) addressing asthma, cancer, cardiovascular disease (CVD), diabetes, mental health; or (5) projects with no clear disease focus, but studying matters of access, efficiency, quality and/or equity in healthcare. The projects included in the first step were then retrieved and individually screened to filter out projects not directly relevant due to no actual use of individual-level data, inappropriate study design, or simply due to lacking information. As a result of the second screening, 34 studies were included in the analysis. A further five studies were included after conversations with public health experts (COMPARE, EPSILON, GBD, Monica, and the OECD Study; see Table 1 for full names). Our final analysis therefore included 39 projects.

## Results

The selection of final projects and inherent websites is displayed in Table 1. To capture the distribution of all projects and their distribution across associated partner countries, an overview of participation levels is provided in Additional file 1. Accordingly, Germany, Spain, France, Italy, Netherlands, and the United Kingdom were the EU countries participating in most projects (at least in 28), compared to e.g. Cyprus, Czech Republic, Latvia, Luxembourg and Slovakia, which participated in the least (maximum of 8). These results are supported by previous research by Galsworthy and McKee [7], who found that the original 15 member states had received 34 times more health research funding under FP7 than the 12 (now 13) newest members.

**Table 1:** List of projects and associated websites

Project	Website (as of December 2016)
B.I.R.O. (started in 2005) (Building a Shared European Diabetes Information System)	www.biro-project.eu/home.htm
COMPARE (initiated in 2006)	No project website available
De-Plan (2005–2008) (Diabetes in Europe – Prevention using Lifestyle, Physical Activity and Nutritional intervention)	www.uniklinikum-dresden.de/de/das-klinikum/kliniken-polikliniken-institute/mk3/klinische-abteilungen/pravention/eu-projekte
DUQuE (2009–2014) (Deepening our understanding of quality improvement in Europe)	www.duque.eu/
ECHIM (2009–2012) (European Community Health Indicators and Monitoring)	www.ec.europa.eu/health/indicators/indicators/index_en.htm
ECHO (2010–2013) (European Collaboration for Health Optimization Project)	www.echo-health.eu/?site
ECRHS III (ongoing project initiated in 2010) (European Community Respiratory Health Survey III)	www.ecrhs.org/ECRHSIII.htm
EPIC (initiated in 1992) (European Prospective Investigation into Cancer and Nutrition)	http://epic.iarc.fr/
EPIC-CVD (2012–2015) (Individualized CVD risk assessment across Europe)	www.epiccvd.eu/
EPIC-Elderly (2002–2005) (The role of diet on the longevity of elderly Europeans)	http://epic.iarc.fr/research/healthyagingepicelderly.php
EPSILON (1996–2000) (A study of care for people with schizophrenia in five European centres)	No project website available
EROS (2002–2005) (The use of stroke registers to assess the quality of stroke management across Europe)	No project website available
ESAW (2002–2004) (European Study of Adult Well-Being)	No official project website available, only a German one: http://www.univie.ac.at/ESAW/
EUBIROD (started in 2008) (EUropean Best Information through Regional Outcomes in Diabetes)	www.eubirod.eu
EUNICE (2006–2008) (European Network for Indicators on Cancer)	No project website available
EUPHORIC (2004–2008) (EU Public Health Outcome Research and Indicators Collection)	No project website available
EUPrimeCare (2010–2012) (Quality and Cost of Primary Care in Europe)	www.eski.hu/new3/kutatas_en/Euprimecare_en.php
EurHOBOP (2009–2011) (European Hospital Benchmarking by Outcomes in Acute Coronary Syndrome Processes)	No project website available
EuroCARE Project (1978–2007) (European Cancer Registry-based study)	www.eurocare.it/
EUROCHIP-3 (2008–2011) (European Cancer Health Indicator Project)	www.tumori.net/eurochip/
EUROCISS (2000–2007) (European Cardiovascular Indicators Surveillance Set)	www.cuore.iss.it/eurociss/en/project/project.asp
EuroDRG (2009–2011) (Diagnosis-Related Groups in Europe: towards Efficiency and Quality)	www.eurodrg.eu
EuroHOPE (2010–2014) (European Health Care Outcomes, Performance and Efficiency)	www.eurohope.info/
EuroTHINE (2004–2007) (Tackling Health Inequalities in Europe)	www.irdes.fr/EspaceAnglais/International/Eurothine.html
Ga2LEN (2004–2009) (Global Allergy and Asthma European Network)	www.ga2len.net/
GBD (2007–2010) (Global Burden of Diseases, Injuries, and Risk Factors Study)	www.healthmetricsandevaluation.org/GBD
HAEMACARE (2005–2008) (Cancer Registry Based project on Haematologic malignancies)	www.haemacare.eu Project website not accessible
HALE (2001–2004) (Healthy Ageing: a Longitudinal study in Europe)	No project website available. Final report available on: www.rivm.nl/bibliotheek/rapporten/260853003.pdf
I2SARE (1999–2007) (Health indicators in the European regions)	www.isare.org/
ISAAC (initiated in 1991) (International Study of Asthma and Allergies in Childhood)	www.isaac.auckland.ac.nz/index.html
JA EHLEIS (2011–2014) (Advanced research on European health expectancies)	www.eurohex.eu/index.php?option=welcome
Monica (initiated in the early 1980s) (Multinational MONItoring of Trends and Determinants in CArdiovascular Disease)	www.thl.fi/monica/
OECD Study (initiated in 2003) (of Cross-National Differences in the Treatment, Costs and Outcomes of Ischaemic Heart Disease)	No project website available. Information available on: http://www.oecd-ilibrary.org/social-issues-migration-health/oecd-study-of-cross-national-differences-in-the-treatment-costs-and-outcomes-of-ischaemic-heart-disease_230112362071
ONCOPOOL (2002–2004) (Pooling of European Data to Harmonize Translational Research in Breast Cancer)	No project website available
PDCAAE (2000–2003) (Prevalence and determinants of childhood asthma and allergies across Europe)	No project website available
QUALICOPC (2010–2013) (Evaluating primary care in Europe)	www.nivel.nl/en/qualicopc
RARECARE (2007–2010) (Surveillance of rare cancers in Europe)	www.rarecare.eu/
SHARE project (initiated 2004) (Survey of Health, Ageing and Retirement in Europe)	www.share-project.org/
WMH Survey Initiative (World Mental Health)	www.hcp.med.harvard.edu/wmh/index.php

A comprehensive overview on essential project information such as ‘governance’, ‘output and results’, or ‘access to data’ can be found on the initially mentioned Health Data Navigator by clicking on the respective project.

**Fig. 1:**
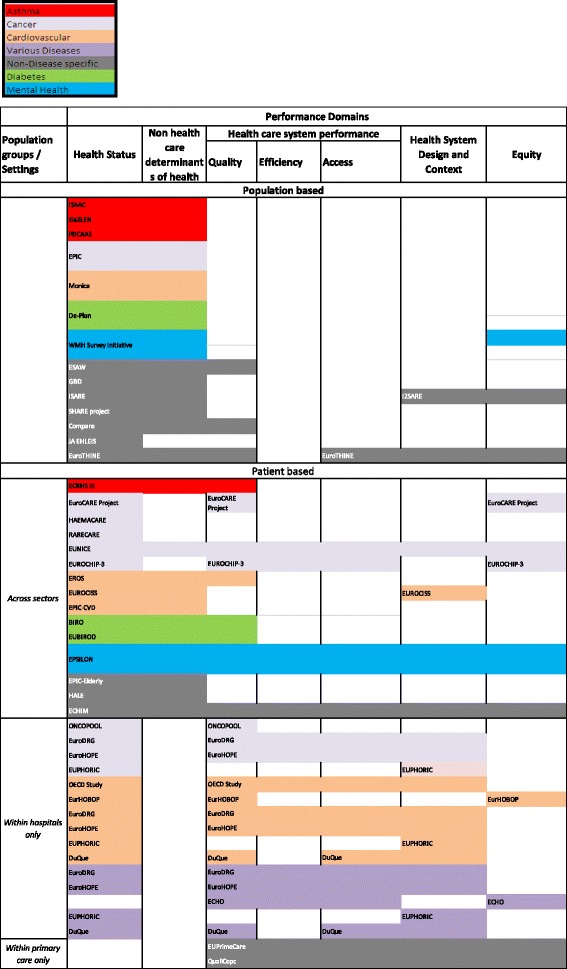
Distribution of EU projects across performance domains; classified by population groups and care settings and disease fields

In respect to research question 1, Fig. 1 indicates the distribution of EU projects across performance domains. In order to systematically analyse the project information and indicate data gaps, all projects were firstly mapped onto the appropriate health system performance domains, and additionally subdivided by care sector, disease field and targeted population group (Fig. 1). The performance domains refer to the OECD Health Care Quality Indicators Framework, a methodological health system performance assessment framework, which was modified to meet the needs of the EuroREACH project of highlighting the production process [9]. Furthermore, to obtain a comprehensive overview on their distribution, all projects were distinguished by whether they target whole populations, i.e. including healthy individuals, or only patients, i.e. diseased individuals. For the latter, we further divided projects by whether they were cross-sectional or not. The disease field dimension was also incorporated into the figure by highlighting projects in different colours (disease fields and corresponding colours are presented in the key below Fig. 1).

### Population-based projects

Projects targeting whole populations are unevenly distributed across performance domains (Fig. 1). The majority of projects only cover the domains ‘health status’ and ‘non-healthcare determinants of health’, with a few exceptions also looking at ‘healthcare system performance’, ‘health system design and context’ and ‘equity’. This might, however, be due to the fact that population-based projects rather assess and compare health statuses by, for example, analysing different lifestyles, intervention starting points, etc. to investigate risk factors for certain conditions (e.g. EPIC) or their impact on population health (e.g. GBD), as well as address healthy ageing (e.g. SHARE) and/or tackle inequality (EuroTHINE), than targeting health services or their performance. In respect to the disease fields covered, most of the population-based projects can be mapped to non-disease specific projects.

### Patient-based projects

Patient-based projects show a better balanced distribution across performance domains, including areas such as quality, efficiency or access to healthcare. We identified projects (e.g. EUNICE) covering all performance domains, whereas others (e.g. RARECARE) cover only one. Among the specific healthcare performance domains, quality was researched most, and efficiency least.

Most projects addressed the hospital and primary care sector and were mapped to the category ‘across sectors’, seven projects were mapped to the inpatient sector (which often benchmark hospital performance, study the quality of the care process and/or different treatment methods) and two projects to the outpatient sector. Thus, the number of projects addressing primary care was considerably smaller than the number of projects addressing the hospital sector, which most likely is not limited to just our project sample.

Disease fields most frequently covered are cancer and CVD. Mental health projects are clearly underrepresented, as well as asthma and diabetes, which might partly be due to the only few primary care projects included. Hospital-based projects mostly study cancer and CVD as well as various acute diseases and focus less on diabetes, asthma and mental health, whereas the primary care projects in our sample rather assess and compare primary care systems than specific diseases.

**Fig. 2:**
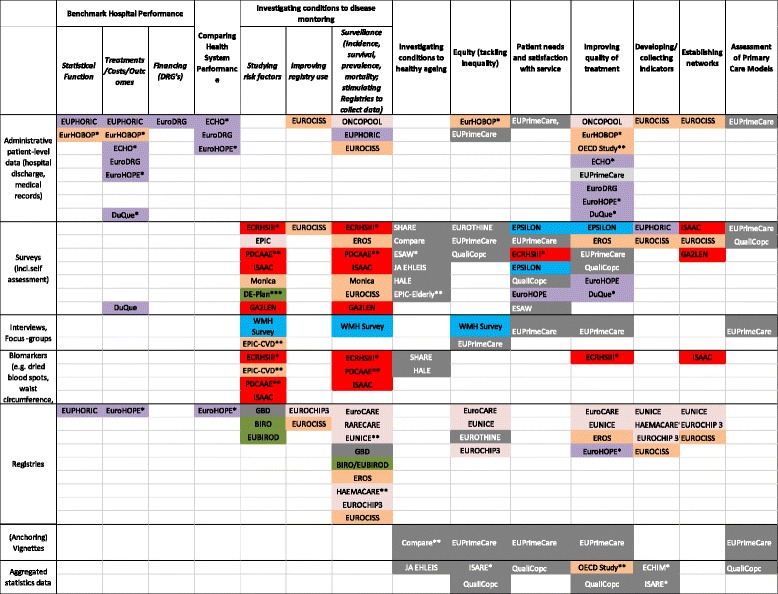
Overview of project objectives and methods

In respect to research question 2, to find out what the projects can tell us about their data and whether there are research imbalances detectable, each project was systematically analysed for details on project objectives, coverage and methods used (Fig. 2). Accordingly, categories for both project objectives and methods were built and populated. The number of projects included in Fig. 2 deviated from those in Fig. 1, since for seven projects the information given on the websites was not sufficient to adequately complete the table.

Regarding project objectives (x-axis), most projects address disease monitoring, healthy ageing, hospital performance and quality of treatment. Data on patient experiences was lacking in most countries, though patient feedback is essential for improving healthcare provision. Information on healthcare costs and health expenditure for particular services and goods is also very poor in these projects, hampering the understanding of health system performance and efficiency. This, however, is often due to the fact that data on costs by diseases and different socioeconomic groups are particularly difficult to obtain for research purposes in many countries.

Thus, two main types of projects exist in the study sample, namely (1) those that monitor health, risk factors or particular diseases across whole populations and develop or adapt indicators but which do not allow an assessment of health system performance (as these dimensions are not included) and (2) those that are often confined to hospital performance, which are patient-level, but necessarily leave out healthy individuals. Fewer projects address equity, patients’ needs and satisfaction or assessed primary care models.

With respect to methods (y-axis), most of the projects in our sample used survey data, administrative patient-level data (i.e. hospital discharge, medical records) and registries. Fewer projects used biomarkers and/or results of a clinical examination (e.g. waist circumference), aggregated statistical data, interviews or focus groups and vignettes. Projects designed for the hospital sector most commonly used patient outcome data, some of which additionally combined different kinds of methods (e.g. EuroHOPE, which combines administrative and survey data, e.g. to assess patient satisfaction, or DuQue, which combines patient outcome data and surveys). It can also be observed that projects addressing CVD, those with no clear disease focus as well as those addressing various diseases were quite wide-spread in their methodology and made use of almost all research instrument, whereas most of the cancer-only projects used registries (Fig. 2).

Figure 2 furthermore indicates follow-up projects in our sample (marked with “*”) which use existing databases for further investigations (e.g. EURHOBOP, which followed EUPHORIC, or ECHIM, which followed ECHI and ECHI II). We also marked collaborative research networks between projects (“**”), thus EUNICE, which operates a network with primary care data providers, collaborates with EuroCARE about their survival registry data and with EUROCHIP about their cancer health indicators. Another example in the field of cancer is RARECARE, a project that helps define indicators and collects data on rare cancers and which collaborates with EUROCARE about their survival registry data, with HAEMACARE on bridging the gap between clinical research and public health information systems, and with EUROCHIP on their indicators.

The following example is to demonstrate the good use of such collection of projects. Using the example of CVD, our hypothetical researcher would theoretically be enabled to compare, for example, risk factors, development of the disease, as well as monitoring and treatment by triangulating research results from EPIC-CVD, Monica, EROS, EUROCISS, EurHOBOP, OECD Study, EuroHOPE and EuroDRG. This kind of performance comparison is, however, still enormously hampered by poor linkage possibilities of data from different providers and sectors (e.g. hospitals, ambulatory care, pharmaceuticals) in many countries as, especially in the field of individual-level health data, many restrictions exist (e.g. in Germany) [9].

## Discussion

The study was driven by the idea to provide a potential user of comparative healthcare information with an inventory of relevant health data across the EU according to their research need (see our inclusion criteria). The first research question, however, already revealed that it is often not easy to find adequate project information. Both of the used information systems did not prove to be entirely sufficient in providing information needed to obtain a proper understanding of the projects’ contents. Deliverables, such as intermediate results, were often not presented or initial project descriptions not updated, even upon project completion. On CORDIS, the quality of information varied considerably between projects; in some cases, projects were presented in detail and also publications listed, in other cases it was not even possible to find a project website or names of project coordinators. Even though FP7 has addressed many of the criticisms of its predecessors [3], comparing our observations to those done in 2009 for FP5 and 6, not much seems to have changed with regard to CORDIS and its difficulties to obtain data.

Once we tried to access individual project websites, the same was often experienced, with user unfriendly, not updated, non-existent or incomplete project websites lacking basic information on project background, methodology and results. An overview of the number of publications listed on each website can be found in Additional file 2. The results reveal large differences between projects and associated publications. In order to obtain a realistic idea about the extent of research funding that is not reflected by the number of publications, a next step should focus on relevant literature databases. Thus, project consortiums should provide complete and easily accessible project websites hosting all relevant information regarding the background, methodology (including sample, data collection process, etc.) and results of the projects (final report, individual outcomes, indicator list) on completion of the study and updates on the progress (e.g. interim reports) when the study is still ongoing. It would be more than unfortunate if good project results disappeared due to poor communication management or because websites were seen as internal communication tools only. Especially funders or governments of under-represented areas might thus be able to spot gaps in existing data, find information on what data have already been processed by others and even use it as a basis or prototype for (local) application and simultaneously be encouraged to collect data in under-represented areas [8]. Ideally, websites would also host the project databases, accessible for researchers or policymakers (e.g. EUROCISS, SHARE) or, as suggested by Galsworthy et al. [10], to have databases generated by projects to be shared mandatorily as project deliverables via central searchable EU repositories. Further, open access to databases at some point after project completion should be allowed for meta-analyses, for example. Initiatives such as the Open Science Prize (https://www.openscienceprize.org/) in the field of biomedical research encourage open content solutions to improve health, which are worth considering in other fields.

In the next step, all projects were carefully analysed to learn about specific research aims and methods used, collaborations developed, and information produced. Overall, we saw that only a few projects had produced the type of data required for proper performance assessment, i.e. that collect not only individual-level data on healthcare performance but also on non-healthcare determinants of health, which impedes proper risk-adjustment. On the other hand, population-based projects, which include such data, almost always lack information on healthcare, i.e. do not allow a performance assessment. This supports previous research indicating that societal needs are not reflected by investments in many funding schemes [11], such as two United Kingdom studies [12, 13] that compared levels of research funding with measures of the burden of disease in the field of infectious diseases as well as in cancer, coronary heart disease, dementia and stroke, and found that funding overall increased within the last years, however, not in relation to their burden of disease. Nevertheless, Horizon 2020 is supposed to address major societal issues such as health and sustainability by, for example, introducing a detailed online hierarchical categorisation of EU investments – similar to the US National Institutes of Health’s RePORTER website – allowing national funders to see collaboration opportunities and gaps [7].

Furthermore, based on our findings, projects should include healthcare utilisation and cost information, which would allow an assessment of the quality of care at different levels (e.g. hospitals, regions, countries). More mixed-method designs would also be useful, for example, cancer projects could incorporate patient discharge data combined with surveys. Additionally, patient-based projects would not only benefit from including non-healthcare determinants, but also from linking their results to cost information (such as EuroHOPE or ECHO) and patient experiences. Other challenges hampering international comparisons are due to a lack of available or accessible data in certain countries and areas, or differences in data collection among countries, e.g. regarding effective care pathways, disparities in healthcare utilisation, patient experience, costs and expenditure, and/or even the most basic descriptive data on socioeconomic inequalities. These observations are partly supported by previous research reporting on research priorities across most fields of public health in European countries [14]. Another issue we came across was the problem of sustainability due to the time-limited construction of the projects and associated funding. Once the projects ended, often, no project website was updated and there was no continued effort to keep the database alive. However, databases generated by projects need to be sustained after completion of projects. In 2015, the BRIDGE Health Project (BRidging Information and Data Generation for Evidence-based Health policy and research; www.bridge-health.eu) was launched to develop a sustainable and integrated EU health information system for both health and research purposes. One of the aims is to continue the work of selected projects relevant for performance assessment, such as ECHO and EuroHOPE.

### Limitations

The aim of this paper was to analyse whether there would be enough research output of EU projects available to conduct proper health system performance. Our approach was to create an option to understand and categorise the output of EU projects by demonstrating a potential solution to systematically analyse them. Since this aim was very ambitious, several limitations were encountered. Inclusion criteria for projects were applied and the search was limited to specific databases only. Thus, relevant projects for health system performance beyond our inclusion criteria and those that were not EU-funded were not considered for the analysis. Furthermore, in CORDIS, only keywords for diseases were used as indicated in the text. Thus, we assumed that, by entering e.g. ‘chronic obstructive pulmonary disease’ instead of COPD, no loss of study information would be implied. Another limitation refers to the approach of assessing performance using projects that were not explicitly funded for performance evaluation. However, we assumed that the overall goal of health-related research should be to improve performance of health systems.

## Conclusions

This study has indicated that public money spent on health research projects and initiatives has produced a huge amount of useful output. At the same time, however, individual projects do not deliver the breadth of information that is necessary for proper health system performance assessment and international comparisons. Therefore, our findings suggest to strengthen the effort of developing datasets that harmonise data from national health surveys and national mortality registries, for example, to allow international comparisons (e.g. EUROTHINE, ONCOPOOL), as well as to implement European-wide benchmarking on outcomes, quality and costs enabling decision-makers as well as health professionals at different levels to learn from the best practices. Additionally, the findings strongly recommend that funders should take clear responsibility not only to make research papers and reports adequately available, but also to ensure that results are made known to the research community, policymakers and funders. CORDIS, as a research and development information service, needs to strengthen its effort to ensure that relevant and detailed project information is adequately shared.

## Additional files

Additional file 1: List of projects and associated partner countries. (DOCX 55 kb)
Additional file 2: List of publications found on project websites. (DOCX 24 kb)
