# Supplementary material for: Is there enough research output of EU projects available to assess and improve health system performance? An attempt to understand and categorise the output of EU projects conducted between 2002 and 2012
Source: Health Res Policy Syst. 2017 Feb 22;15:13. doi: 10.1186/s12961-016-0165-5 (PMC5322637; doi:10.1186/s12961-016-0165-5)
Supplement: Additional file 1: — List of projects and associated partner countries. (DOCX 55 kb) [file 12961_2016_165_MOESM1_ESM.docx]

Annex 1: List of projects and associated partner countries

| * GBD project covers worldwide data for 187 countries | | | | | | | | |  |  |  |  |  |  |  |  |  |  |  |  |  |  |  |  |  |  |  |  |  |  |  |  |  |  |  |  |  |  |  |  |  |  |  |  |  |  |  |  |  |  |  |  |
| --- | --- | --- | --- | --- | --- | --- | --- | --- | --- | --- | --- | --- | --- | --- | --- | --- | --- | --- | --- | --- | --- | --- | --- | --- | --- | --- | --- | --- | --- | --- | --- | --- | --- | --- | --- | --- | --- | --- | --- | --- | --- | --- | --- | --- | --- | --- | --- | --- | --- | --- | --- | --- |
| **ISAAC covers data from 105 countries | | | | |  |  |  |  |  |  |  |  |  |  |  |  |  |  |  |  |  |  |  |  |  |  |  |  |  |  |  |  |  |  |  |  |  |  |  |  |  |  |  |  |  |  |  |  |  |  |  |  |
|  |  |  |  |  |  |  |  |  |  |  |  |  |  |  |  |  |  |  |  |  |  |  |  |  |  |  |  |  |  |  |  |  |  |  |  |  |  |  |  |  |  |  |  |  |  |  |  |  |  |  |  |  |
| *Country key* |  |  |  |  |  |  |  |  |  |  |  |  |  |  |  |  |  |  |  |  |  |  |  |  |  |  |  |  |  |  |  |  |  |  |  |  |  |  |  |  |  |  |  |  |  |  |  |  |  |  |  |  |
| BE= Belgium; BG= Bulgaria; CZ= Czech Republic; DE= Germany; DK=Denmark; EE= Estonia; IE= Ireland; EL= Greece; ES= Spain; FR= France; IT= Italy; CY= Cyprus; LV= Latvia; LU= Lithuania; LU= Luxembourg; HU= Hungary; MT= Malta; NL= Netherlands; AT= Austria; PL= Poland; PT= Portugal; RO= Romania; SI= Slovenia; SK= Slovakia; FI= Finland; SE= Sweden; UK= United Kingdom; HR= Croatia; ME= Montenegro; IS= Iceland; RS= Serbia; TR= Turkey; NO= Norway; CH= Switzerland; AL= Albania; MD= Moldova; KV= Kosovo; FYROM= Former Yugoslav Republic of Macedonia; UA= Ukraine; IL= Israel; RUS= Russia; AU= Australia; NZ= New Zealand; JP= Japan; USA= United States of America; CAN= Canada; MX= Mexico; BR= Brazil; PE= Peru; CO= Colombia; CR= Costa Rica; NG= Nigeria; ZA= South Africa; LB= Lebanon; IQ= Iraq; KW= Kuwait; VN= Vietnam; KR= Republic of Korea; SG= Singapore; PRC= People's Republic of China; IN= India | | | | | | | | | | | | | | | | | | | | | | | | | | | | | | | | | | | | | | | | | | | | | | | | | | | | |
|  |  |  |  |  |  |  |  |  |  |  |  |  |  |  |  |  |  |  |  |  |  |  |  |  |  |  |  |  |  |  |  |  |  |  |  |  |  |  |  |  |  |  |  |  |  |  |  |  |  |  |  |  |
|  |  |  |  |  |  |  |  |  |  |  |  |  |  |  |  |  |  |  |  |  |  |  |  |  |  |  |  |  |  |  |  |  |  |  |  |  |  |  |  |  |  |  |  |  |  |  |  |  |  |  |  |  |
|  |  |  |  |  |  |  |  |  |  |  |  |  |  |  |  |  |  |  |  |  |  |  |  |  |  |  |  |  |  |  |  |  |  |  |  |  |  |  |  |  |  |  |  |  |  |  |  |  |  |  |  |  |
|  |  |  |  |  |  |  |  |  |  |  |  |  |  |  |  |  |  |  |  |  |  |  |  |  |  |  |  |  |  |  |  |  |  |  |  |  |  |  |  |  |  |  |  |  |  |  |  |  |  |  |  |  |
