# Supplementary material for: Is there enough research output of EU projects available to assess and improve health system performance? An attempt to understand and categorise the output of EU projects conducted between 2002 and 2012
Source: Health Res Policy Syst. 2017 Feb 22;15:13. doi: 10.1186/s12961-016-0165-5 (PMC5322637; doi:10.1186/s12961-016-0165-5)
Supplement: Additional file 2: — List of publications found on project websites. (DOCX 24 kb) [file 12961_2016_165_MOESM2_ESM.docx]

**Annex 2: List of publications found on project websites**

| Project | Website (as of January 2015) |
| --- | --- |
| B.I.R.O. (started in 2005) | Journal Articles: 1, book chapters: 2, other project output (excluding journal articles & book chapters): 7 |
| COMPARE (initiated in 2006) | No project website available |
| De-Plan (2005-2008) | No project website available |
| DUQuE (2009-2013) | Journal articles: 3, other project output: 3 |
| ECHIM (2009-2012) | No publications listed |
| ECHO (2010-2013) | No publications listed |
| ECRHS III (ongoing project initiated in 2010) | Journal articles & books: 280 |
| EPIC (initiated in 1992) | Journal articles: 4 (Website refers to WHO database for further articles) |
| EPIC-CVD (2012-2015) | Journal articles: 2, part of the EPIC study |
| EPIC-Elderly (2002-2005) | Website does not list publications, but refers to WHO database |
| EPSILON (1996-2000) | EPSILON (1996-2000) |
| EROS (2002-2005) | No project website available |
| ESAW (2002-2004) | Journal articles: 1, book chapter: 1, other project output: 100+ |
| EUBIROD (started in 2008) | Books: 1, journal articles: 2, other project output: 10 |
| EUNICE (2006-2008) | No project website available |
| EUPHORIC (2004-2008) | Journal articles: 5, other project output: 31 |
| EUPrimeCare (2010-2012) | No publications listed |
| EurHOBOP (2009-2011) | Journal articles: 2 |
| EuroCARE Project (1978-2007) | Journal articles & books: 133 |
| EUROCHIP-3 (2008-2011) | Journal articles: 26, book chapters: 5, other project output: 21 |
| EUROCISS (2000-2007) | No publications listed |
| EuroDRG (2009 -2011) | Journal articles: 13, book chapters: 23, other project output: 41 |
| EuroHOPE (2010-2014) | Journal articles: 4, other project output: 6 |
| EuroTHINE (2004-2007) | Project website not accessible |
| Ga2LEN (2004-2009) | Journal articles: 42, other project output: 10 |
| GBD (2007-2010) | Journal articles: 50, other project output: 10 |
| HAEMACARE (2005-2008) | Project website not accessible www.haemacare.eu |
| HALE (2001-2004) | No project website available |
| I2SARE (1999-2007) | No publications listed |
| ISAAC (initiated in 1991) | Journal articles: 540, other project output: 83 |
| JA EHLEIS (2011-2014) | Journal articles: 14, book chapters: 4, other project output: 88 |
| Monica (initiated in the early 1980s) | Journal articles: 110, books: 1 |
| OECD Study (initiated in 2003) | No project website available |
| ONCOPOOL (2002-2004) | No publications listed |
| PDCAAE (2000-2003) | No project website available |
| QUALICOPC (2010-2013) | Journal articles: 10 |
| RARECARE (2007-2010) | Journal articles: 28, other project output: 16 |
| SHARE project (initiated 2004) | Journal Articles: 471, books: 62, book chapters: 228 |
| WMH Survey Initiative | Journal articles & books: 667 (In Press: 8) |
